# Cardiac Rhythm Monitoring After Acute Decompensation for Heart Failure: Results from the CARRYING ON for HF Pilot Study

**DOI:** 10.2196/resprot.4380

**Published:** 2016-04-26

**Authors:** Emilio Vanoli, Andrea Mortara, Paolo Diotallevi, Giuseppe Gallone, Barbara Mariconti, Edoardo Gronda, Alessandra Gentili, Silvia Bisetti, Giovanni Luca Botto

**Affiliations:** ^1^Dept Molecular Medicine, University of Pavia, ItalySesto San Giovanni, Milano,Italy; ^2^Policlinico di MonzaCardiovascular DepartmentMonzaItaly; ^3^Clinica San GaudenzioNovaraItaly; ^4^Azienda Ospedaliera S.AnnaComoItaly; ^5^IRCCS MultimedicaSesto San Giovanni, MilanoItaly; ^6^Medtronic Regional Clinical CenterRomaItaly; ^7^Medtronic ItaliaMilanoItaly

**Keywords:** continuous cardiac monitoring, implantable loop recorder, acute heart failure, arrhythmias

## Abstract

**Background:**

There’s scarce evidence about cardiovascular events (CV) in patients with hospitalization for acute heart failure (HF) and no indication for immediate device implant.

**Objective:**

The CARdiac RhYthm monitorING after acute decompensatiON for Heart Failure study was designed to assess the incidence of prespecified clinical and arrhythmic events in this patient population.

**Methods:**

In this pilot study, 18 patients (12 (67%) male; age 72±10; 16 (89%) NYHA II-III), who were hospitalized for HF with low left ventricular ejection fraction (LVEF) (<40%) and no immediate indication for device implant received an implantable loop recorder (ILR) before hospital discharge. Follow-up visits were scheduled at 3 and 6 months, and at every 6 months until study closure; device data were remotely reviewed monthly. CV mortality, unplanned CV hospitalization, and major arrhythmic events during follow-up were analyzed.

**Results:**

During a median follow-up of 593 days, major CV occurred in 13 patients (72%); of those, 7 patients had at least 1 cardiac arrhythmic event, 2 had at least a clinical event (CV hospitalization or CV death), and 4 had both an arrhythmic and a CV event. Six (33%) patients experienced 10 major clinical events, 5 of them (50%) were HF related. During follow-up, 2 (11%) patients died due to a CV cause and 3 (16%) patients received a permanent cardiac device.

**Conclusions:**

After an acute HF hospitalization, patients with LVEF<40% and who are not readily eligible for permanent cardiac device implant have a known high incidence of major CV event. In these patients, ILR allows early detection of major cardiac arrhythmias and the ability to react appropriately in a timely manner.

**Trial Registration:**

ClinicalTrials.gov NCT01216670; https://clinicaltrials.gov/ct2/show/NCT01216670

## Introduction

Acute heart failure (AHF) episodes that induce hospitalizations represent one of the largest causes of health status deterioration [1]. About 45% of patients hospitalized with AHF will be rehospitalized at least once (and 15% at least twice) within 12 months [2]. Estimates of the risk of death or rehospitalization within 60 days of admission vary from 30% to 60%, depending on the population studied [2-8]. A significant proportion of heart failure (HF) patients do not undertake any device implant strategy since they do not meet the guidelines criteria [2]. Very little, if any, monitored information exists in those patients experiencing AHF with modest left ventricle impairment or with HF with preserved left ventricular ejection fraction (LVEF). Timely information might allow interventions that then avoid major cardiovascular (CV) events and possible progressive worsening of the disease leading to hospitalization. The availability of implantable devices to continuously monitor cardiac trends may provide early warning of changes in cardiac status, which would then allow early clinical management and possibly reduce the number of HF hospitalizations. The CARdiac RhYthm monitorING after acute decompensatiON for Heart Failure (CARRYING ON for HF) trial was designed to assess the efficacy of implantable loop recorders (ILRs) in the early detection of prespecified clinical and arrhythmic events in this patient population.

## Methods

### Study Design and Patient Population

In this prospective pilot study, patients who were hospitalized for AHF, had an LVEF <40% but no immediate indication for device implant, and received an ILR before hospital discharge were enrolled.

**Figure 1:**
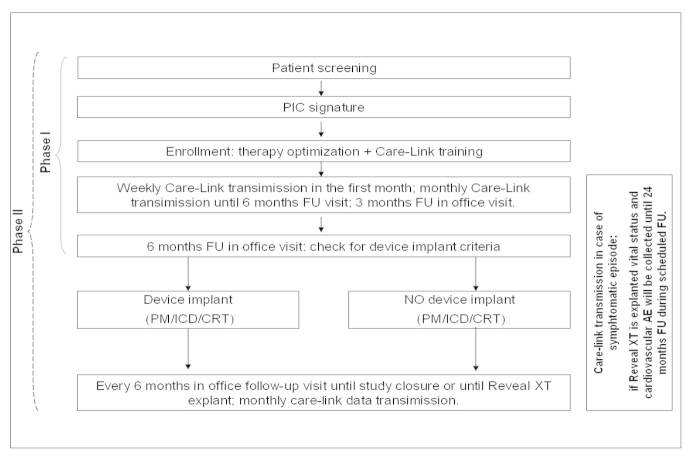
Study design.

Clinical follow-up visits were scheduled at 3 and 6 months and every 6 months thereafter. Device data were reviewed monthly through the Care-Link remote monitoring system or at any time the patients had symptoms (ClinicalTrials.gov; Identifier: NCT01216670). CARRYING-ON for HF Study design is reported in Figure 1.

Informed consent was obtained from each enrolled patient and the study protocol conforms to the ethical guidelines of the 1975 Declaration of Helsinki as reflected in a priori approval by the local ethics committee.

The aim of the study was to assess the capability of loop recording to recognize early signs of prespecified clinical and arrhythmic events in this cohort of patients. Prespecified events were: (1) CV mortality or unplanned CV hospitalization and (2) any cardiac arrhythmic event detected by the implanted device (Sinus bradycardia: ≤ 30 beats per minutes (bpm), ≥ 8s; Sinus arrest: ≥ 5s; atrioventricular (AV) block (2°, 3°): ≤ 30 bpm, ≥ 8s; atrial fibrillation (AF): > 6 min; non-sustained ventricular tachycardia (VT): ≥ 125 bpm, ≥16 beats; sustained VT: > 30 sec). The main inclusion criteria were: (1) a history of at least 1 HF hospitalization, emergency department visit, or urgent office visit necessitating intravenous (IV) diuretic or augmentation of oral diuretic, IV inotropic, or IV vasodilator or other HF parenteral therapy within 15 days prior to device implant; (2) an implanted Medtronic Reveal XT ILR device (< 15 days post-implant); and (3) LVEF < 40%. Main exclusion criteria were: (1) New York Heart Association (NYHA) Class IV (chronic or ambulatory); (2) planned or previous implant of an implantable cardioverter defibrillator (ICD) or pacemaker device; (3) severe chronic obstructive pulmonary disease; (4) permanent AF at time of enrolment; and (5) ST segment elevation at electrocardiogram (ECG). A Reveal XT ILR was used for continuous cardiac monitoring. The ILR was implanted within 15 days the AHF event. All the patients enrolled received a remote monitoring system and were requested to transmit Reveal XT data on a monthly base or in case any symptoms occurred.

### Statistical Analysis

Continuous data were summarized as mean and standard deviation or median and 25th-75th percentiles in case of skewed distributions. Absolute and relative frequencies were reported for categorical variables. Kaplan-Meier method was used to display the time to first event. The mean cumulative function (MCF) plot for the number of recurrent events was used to display the overtime trend of the events rate. Statistical analyses were performed using SAS 9.3 for Windows (SAS Institute Inc, Cary, NC).

## Results

### Baseline Characteristics

The patients on average were 73±10 years old, 12 (67%) were male, and 7 (39%) had ischemic cardiomyopathy. According to guidelines, all the patients at the time of enrollment had no immediate indication to receive a permanent implantable cardiac device: 8 (44%) patients had LVEF > 35%, 2patients were in NYHA functional class I, and 9 (50%) patients were not under optimized pharmacological treatment for HF at the time of the AHF event. Most patients (15/18, 83%) were on beta-blocker therapy (11 were on bisoprolol 2.5 mg twice a day and 4 were on carvedilol 12.5 mg twice a day). Further baseline characteristics are reported in Table 1.

**Table 1:** Baseline characteristics of study population.

Baseline Patients Characteristics^a^		N=18 (%)
Age, y		73±10
Gender	Male	12 (67%)
Ischemic etiology		7 (39%)
History of AF		8 (44%)
CHADS_2_ score (for AF patients)		3.0+0.7
History of NS-VT		2 (18%)
QRS complex, ms		106±23
LBBB		4 (22%)
		
NYHA	Functional class I	2 (11%)
	Functional class II	13 (72%)
	Functional class III	3 (17%)
		
Cardiovascular-Related Diseases	MI + CAD	6 (35%)
	Hypertension	11 (65%)
	Valvular heart disease	6 (35%)
	Previous cardiovascular surgeries	9 (50%)
	Type 2 diabetes	4 (22%)
		
		
Echocardiographic Measurements	LVEF (%)	34±6.0
	LVEF ≤ 35%	10 (56%)
	LVESV, mL	108±38
	LVESV ≥ 100 mL	7 (47%)
		
Drug Therapy at Discharge	Diuretic	17 (94%)
	ACE inhibitor and/or ARB	16 (89%)
	Beta blocker	15 (83%)
	Amiodarone	10 (56%)
	Anticoagulant	14 (78%)

Abbreviations: ACE, angiotensin-converting enzyme; AF, atrial fibrillation; ARB, angiotensin receptor blocker; CAD, coronary artery disease; LBBB, left bundle branch block; LVEF, left ventricular ejection fraction; LVESV, left ventricular end-systolic volume; MI, myocardial infarction; NS-VT, non-sustained ventricular tachycardia; NYHA, New York Heart Association.

^a^Data are expressed as mean ± standard deviation or absolute (relative) frequencies.

### Clinical Outcomes

**Figure 2:**
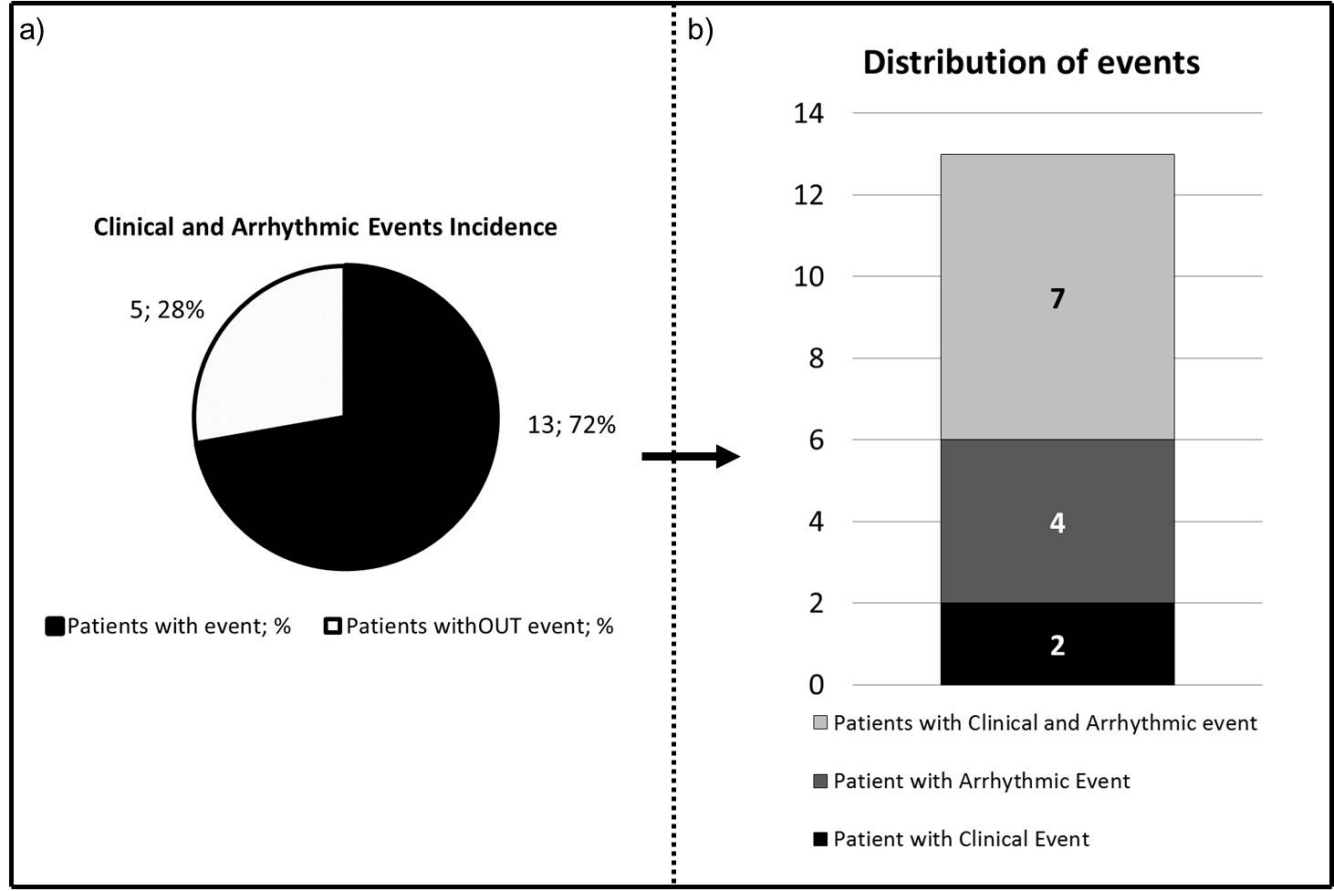
Clinical and arrhythmic events incidence and distribution.

During a median follow-up of 593 days (mean 509+260), major CV events occurred in 13 patients (72%, median time to first combined event 162 days (39-606)); 11 patients had at least 1 cardiac arrhythmic event, 2 had at least 1 clinical event (CV hospitalization or CV death), and 4 had both arrhythmic and clinical events. Figure 2 shows clinical and arrhythmic events incidence and distribution.

Six (33%) patients experienced 10 major clinical events; 5 (50%) of which were HF-related with 1 patient dying due to HF (terminal rhythm: asystole) and 1 patient dying due to an acute CV event (ruptured cerebral aneurysm). Distribution of detected major clinical and arrhythmic events is reported in Table 2.

**Table 2:** Type and frequency of clinical and arrhythmic events that occurred^a^.

Clinical Events		Events (n=10)	Patients with Events (n=6)
CV death^b^, n (%)		2 (20%)	2 (33%)
	of which HF-related death, n (%)	1 (10%)	1 (1.7%)
CV hospitalizations, n (%)		10 (100%)	6 (100%)
	of which HF-related hospitalizations, n (%)	5 (50%)	3 (50%)
			
Arrhythmic Events		Events (n=1326)	Patients with Events (n=11)
	Synus bradycardia, n (%) (≤ 30 bpm, ≥ 8s)	8 (0.6%)	3 (27%)
	Sinus arrest, n (%) (≥ 5s)	2 (0.2%)	2 (18%)
	AV block, n (%) (≤ 30 bpm, ≥ 8s)	--	--
	AF-AT, n (%) (> 6 min)	1297 (97.7%)	8 (73%)
	Non-Sustained VT, n (%) (≥ 125 bpm, ≥16 beats)	13 (1%)	2 (18%)
	Sustained VT, n (%) (> 30 sec)	6 (0.5%)	2 (18%)

Abbreviations: AF, atrial fibrillation; AT, atrial tachycardia; AV, atrioventricular; CV, cardiovascular; HF, heart failure; VT, ventricular tachycardia.

^a^Data are expressed as absolute (relative) frequencies.

^b^The 2 events of death also had previous hospitalizations.

During follow-up, 3 (16%) patients were implanted with a permanent cardiac device according to current guidelines (in 2 patients who developed sustained-VT, a cardiac resynchronization therapy with defibrillator backup device (CRT-D) was implanted; in the 1 patient with sinus arrest and LVEF < 35%, a single chamber ICD was implanted). Eight patients suffered paroxysmal AF that was often asymptomatic (62%); the mean CHADS_2_ score in these 8 patients was 3.0+0.7, as all had a CHADS_2_ score ≥2.

As per heart rate (HR), we computed its circadian behavior: the 2 patients who had a nocturnal HR > 70 bpm for > 85% of the night had major clinical events, while this occurred in only 3/16 (19%) of the patients with nocturnal HR < 70 bmp.

**Figure 3:**
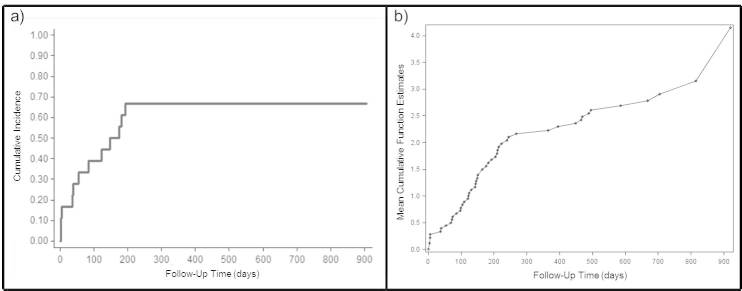
Kaplan-meier analysis for clinical and arrhythmic events.

Figure 3 shows Kaplan-Meier analysis for clinical and arrhythmic events, both for time to first combined event (Figure 3a) and for cumulative combined events incidence (Figure 3b).

## Discussion

The main finding from this study, which might carry significant clinical implications, is that continuous ECG and HR monitoring by loop recorders might contribute to the early recognition of cardiac rhythm abnormalities contributing to an observed very high incidence of major CV (72%) events. Such high event risk is known to occur in patients with LVEF < 40% who are not eligible for ICD implant within 6 months after an HF hospitalization. The study population, despite its small size, was representative of the clinical reality: elderly patients, prevalently NYHA class II, with a mean CHADS_2_ score of 3. The high morbidity detected in the study was not unexpected, but the clinical information provided by the CARRYING ON for HF study deserves specific attention. We can conjecture that the appropriate use of remote monitoring as part of a more comprehensive approach to such patients may decrease the observed high re-hospitalization rate after an AHF. The CARRYING-ON HF study was designed to record events but did not prescribe any specific intervention. It is, however, reasonable to believe that the early detection of rhythm and conduction disturbances by ILR will allow clinicians to react appropriately in a more timely manner, avoiding quick progression of the disease and worst outcomes.

In fact, half of the study population had an AHF during the study period. These patients were being treated with a suboptimal HF medical strategy and the continuous monitoring led to subsequent optimal medical titration for HF, arrhythmia management, and stroke prevention. This should be understood in view of the CRYSTAL AF study [9] indicating that stroke may be the first clinical manifestation of AF. The analysis of the time to first combined event (Figure 3) and of the cumulative combined events provides further important information. The first is that a 6-month-long monitoring period is not sufficient to detect a first significant event in all the patients who are bound to have them. Secondly, beyond 6 months a small proportion of combined events occurred possibly because of the fatal event on one side and the optimization of patient management induced by the monitoring device on the other. In this context it is noteworthy that 16/18 (89%) patients had an optimal nocturnal HR (less < 70 bpm). Thus, it may not be incidental that the only 2 patients with an overnight inappropriate HR had clinical events while this occurred in only 3/16 (19%) patients with controlled nocturnal HR. This observation is indeed coherent with the predictive value of HR and its circadian variation [10-11]. Furthermore the ILR detected episodes of sustained VT that met the criteria for 2 CRT-D implants. In a third case, a detection of a life-threatening event occurred: nocturnal long pauses (> 8 sec) were discovered. In this patient, the concomitant presence of LVEF < 35% and the AV conduction disturbance led to a single chamber ICD implant. These 3 cases were diagnosed by the ILR and allowed the timely intervention that prevented a potential life-threatening event.

### Limitations

The main limitation of this pilot study is the small number of patients enrolled. Our study, planned as a feasibility and methodology study, was not powered to determine the overall prevalence of CV events after an AHF episode nor to test the effectiveness of ILR in reducing CV events in this population. The primary aim was descriptive as a preliminary fundamental information to design a larger comparative study.

### Conclusions

The CARRYING ON for HF study provided meaningful information on the many (72%) patients hospitalized for an acute HF episode who are not eligible, according to current guidelines, to receive an implantable cardiac device and who later develop CV and arrhythmic events during the follow-up.

The present finding provides some background for future prospective studies aimed at the assessment of the risk stratification of continuous cardiac monitoring in HF patients who are not deemed eligible for immediate implantation of a permanent cardiac device.

## Abbreviations

ACE: angiotensin-converting enzyme
AF: atrial fibrillation
AHF: acute heart failure
ARB: angiotensin receptor blocker
AV: atrioventricular
bpm: beats per minutes
CAD: coronary artery disease
CARRYING ON for HF: The CARdiac RhYthm monitorING after acute decompensatiON for Heart Failure
CRT-D: cardiac resynchronization therapy with defibrillator back-up device
CV: cardiovascular
ECG: electrocardiogram
HF: heart failure
HR: heart rate
ICD: implantable cardioverter defibrillator
ILR: implantable loop recorder
IV: intravenous
LBBB: left bundle branch block
LVEF: left ventricular ejection fraction
LVESV: left ventricular end-systolic volume
MI: myocardial infarction
NS-VT: non-sustained ventricular tachycardia
NYHA: New York Heart Association
VT: ventricular tachycardia
